# Mapping of a major QTL for increased robustness and detection of genome assembly errors in Asian seabass (*Lates calcarifer*)

**DOI:** 10.1186/s12864-023-09513-z

**Published:** 2023-08-10

**Authors:** Xueyan Shen, Yong Chao Niu, Joseph Angelo V. Uichanco, Norman Phua, Pranjali Bhandare, Natascha May Thevasagayam, Sai Rama Sridatta Prakki, László Orbán

**Affiliations:** 1 Reproductive Genomics Group, Temasek Life Sciences Laboratory, Singapore, Singapore; 2 Tropical Futures Institute, James Cook University Singapore, Singapore, Singapore; 3 Biozeron Shenzhen, Inc., Shenzhen, China; 4 James Cook University Singapore, Singapore, Singapore; 5 Present Address: School of Chemical & Life Sciences, Life Sciences Applied Research Group, Nanyang Polytechnic, Singapore, Singapore; 6 Present address: Theodor Boven Institute (Biocenter), University of Würzburg, Würzburg, Germany; 7 Present address: Infectious Disease Research Laboratory, National Centre for Infectious Diseases, Tan Tock Seng Hospital, Singapore, Singapore; 8 Frontline Fish Genomics Research Group, Department of Applied Fish Biology, Institute of Aquaculture and Environmental Safety, Georgikon Campus, Hungarian University of Agriculture and Life Sciences, Keszthely, Hungary

**Keywords:** Asian seabass, Linkage map, Genome, Quantitative trait loci, Robustness

## Abstract

**Background:**

For Asian seabass (*Lates calcarifer,* Bloch 1790) cultured at sea cages various aquatic pathogens, complex environmental and stress factors are considered as leading causes of disease, causing tens of millions of dollars of annual economic losses. Over the years, we conducted farm-based challenges by exposing Asian seabass juveniles to complex natural environmental conditions. In one of these challenges, we collected a total of 1,250 fish classified as either ‘sensitive’ or ‘robust’ individuals during the 28-day observation period.

**Results:**

We constructed a high-resolution linkage map with 3,089 SNPs for Asian seabass using the double digest Restriction-site Associated DNA (ddRAD) technology and a performed a search for Quantitative Trait Loci (QTL) associated with robustness. The search detected a major genome-wide significant QTL for increased robustness in pathogen-infected marine environment on linkage group 11 (ASB_LG11; 88.9 cM to 93.6 cM) with phenotypic variation explained of 81.0%. The QTL was positioned within a > 800 kb genomic region located at the tip of chromosome ASB_LG11 with two Single Nucleotide Polymorphism markers, R1-38468 and R1-61252, located near to the two ends of the QTL. When the R1-61252 marker was validated experimentally in a different mass cross population, it showed a statistically significant association with increased robustness. The majority of thirty-six potential candidate genes located within the QTL have known functions related to innate immunity, stress response or disease. By utilizing this ddRAD-based map, we detected five mis-assemblies corresponding to four chromosomes, namely ASB_LG8, ASB_LG9, ASB_LG15 and ASB_LG20, in the current Asian seabass reference genome assembly.

**Conclusion:**

According to our knowledge, the QTL associated with increased robustness is the first such finding from a tropical fish species. Depending on further validation in other stocks and populations, it might be potentially useful for selecting robust Asian seabass lines in selection programs.

**Supplementary Information:**

The online version contains supplementary material available at 10.1186/s12864-023-09513-z.

## Background

Asian seabass (*Lates calcarifer,* Bloch 1790), also known as barramundi (from here onwards seabass), is an eminent tropical commercial food fish species. Its farming has originally been developed in the Southeast Asian countries, Australia and Papua New Guinea [1], however, it has also entered the seafood markets of Europe and the United States since then [2]. Regional production of this species in major producing countries increased from ~ 20,000 tons in 1998 to 90,000 tons in 2017 [3]. In Singapore, marine net-cages from small family-owned and mid-sized fish farms (using brooders collected either from the sea or unknown sources) are the most popular method for culturing the species [4].

Currently, the aquaculture production of seabass is hampered by frequent outbreaks of viral, bacterial or parasitic infectious diseases, especially for fish directly exposed to the natural environment. These often drastically reduce the chance for survival of farmed stocks and cause substantial economic losses for the farms. A wide range of pathogens, including Scale Drop Disease Virus (SDDV) [5, 6], *L. calcarifer* Herpes Virus (LCHV) [7], ‘big belly’ bacteria [8], and *Streptococcus iniae* [9], have been detected in cultured seabass and they are a major concern for the present and future sustainability their culture. While serious diseases are often reported in association with specific aquatic pathogens, environmental factors (e.g., pollutants, stress and increased temperature) were also shown to contribute to the formation of disease outbreaks in fish stocks [10–12]. Until now, the understanding of interactions among host, pathogen and environment during infection is lacking in seabass aquaculture, and accordingly, there are also no effective control measures for many of these problems. The identification of markers, genes and pathways involved in the response to disease infection by the fish plays an important role in the generation of robust fish lines with increased disease tolerance. Marker-assisted selection (MAS) through quantitative trait loci (QTL) mapping has become a potential solution to improve disease resistance, helping to improve the performance of cultured farm fish stocks substantially while generating huge economic benefits [13, 14]. To date, many studies on identifying QTLs associated with resistance to bacterial, viral or parasitic disease have been conducted in diverse fish species of great economic values (for review see: [14]). For instance, in rainbow trout, QTL resistant to infectious hematopoietic necrosis virus (IHNV) [15], bacterial cold water disease (BCWD) [16, 17], and *Vibrio anguillarum* [18] were detected on various chromosomes of the species. Most importantly, the QTL with major effects for resistance against infectious pancreatic necrosis virus (IPNV) in Atlantic salmon [19–21] as well as the QTL against lymphocystis disease in Japanese flounder [22] have been applied in selective breeding programs, leading to substantially reduced outbreaks of the disease.

Our former research team – together with our collaborators – has been working on seabass selective breeding for nearly two decades, mainly focusing on the improvement of growth-related traits (for review see: [4]). In addition, several studies on traits potentially associated with increased disease tolerance of seabass have also been conducted using lab-based setups in which fish were challenged with a single pathogen in every experiment. Until now, at least 15 significant QTLs with minor effects associated with increased resistance against nervous necrosis virus (NNV; [23–25]) and another four QTLs against Singapore grouper iridovirus [26] have been detected. In the present study, we have subjected seabass fingerlings to a series of challenges under typical farm conditions (a complex pathogen-infected marine environment), which were expected to have direct relevance to production under commercial conditions. Our main purpose was to identify QTLs (and possibly genes as well as related pathways) associated with increased robustness using high-resolution linkage mapping based on double digest Restriction-site Associated DNA (ddRAD).

High resolution linkage maps based on ddRAD or Single Nucleotide Polymorphism (SNP) chip technologies play an important role in animal genomics by helping the production of chromosomal-level genome assemblies through anchoring and orienting of scaffolds [27–29]. They also facilitate mapping of complex traits for genetic breeding in many economically important aquaculture species (for reviews see e.g.: [30]). In addition, they are also useful tools for validation or improvement of de novo genome assemblies. When the draft genomes of Pacific oyster (*Crassostrea gigas*) [31] and several teleost species (see e.g. [32–34], have been evaluated by high-resolution linkage maps, all of them showed widespread errors in their scaffold assembly. For the seabass, the chromosomal-level genome assembly [35] was generated using PacBio-based long-reads augmented by transcriptomics, optical and low density genetic mapping along with shared synteny from of European seabass (*Diacentrarchus labrax*) and three-spined stickleback (*Gastrosteus aculeatus*). Although the assembly quality of the seabass reference genome (contig & scaffold N50 size: over 1 Mb & 25 Mb, respectively) far exceeded those of most other fish genomes published earlier, it had only 22,184 annotated genes. We hope that a high resolution ddRAD map will help us to validate and further improve the current seabass reference genome assembly.

Our long-term goal was to enhance our seabass stocks and establish elite lines with fast growth, improved fillet yield & flesh quality, and especially increased robustness through MAS as well as genomic selection (GS). This project was designed to achieve advances towards those goals by fulfilling the following specific objectives: 1) constructing a high-density linkage map; 2) identifying QTLs for increased robustness; 3) discovering potential causative candidate genes within those QTL regions and related pathways; and 4) validating the effect of SNPs within the QTL in a mass cross population. An additional goal of the project was to detect mis-assembled sequences and their corresponding chromosomes to further improve the current reference genome assembly [35].

## Results

### Complex field environment challenge

Altogether, ten sea-based farm trials were performed in order to select for families with robust offspring that show a substantially higher percentage of survival than the rest. Seabass fingerlings originating from mass crosses were grown under sheltered conditions for about one month. Then they were transferred into full sea water at 30–60 dph of age and their survival was documented during a 1–4 month of time period (Supplementary Fig. S1 shows the survival curves of three different batches of seabass fingerlings exposed to raw sea water at the same farm). Out of the 47 families tested, 16 (34%) showed a substantial increase in their contribution when the relative proportion of their offspring in their batch at the end of exposure to pathogen-containing sea water was compared to the initial one (see Supplementary Fig. S2 for an example). Eleven of the 35 (31%) brooders tested participated in at least two such families and four of them (11%) contributed to more than two.

**Fig. 1:**
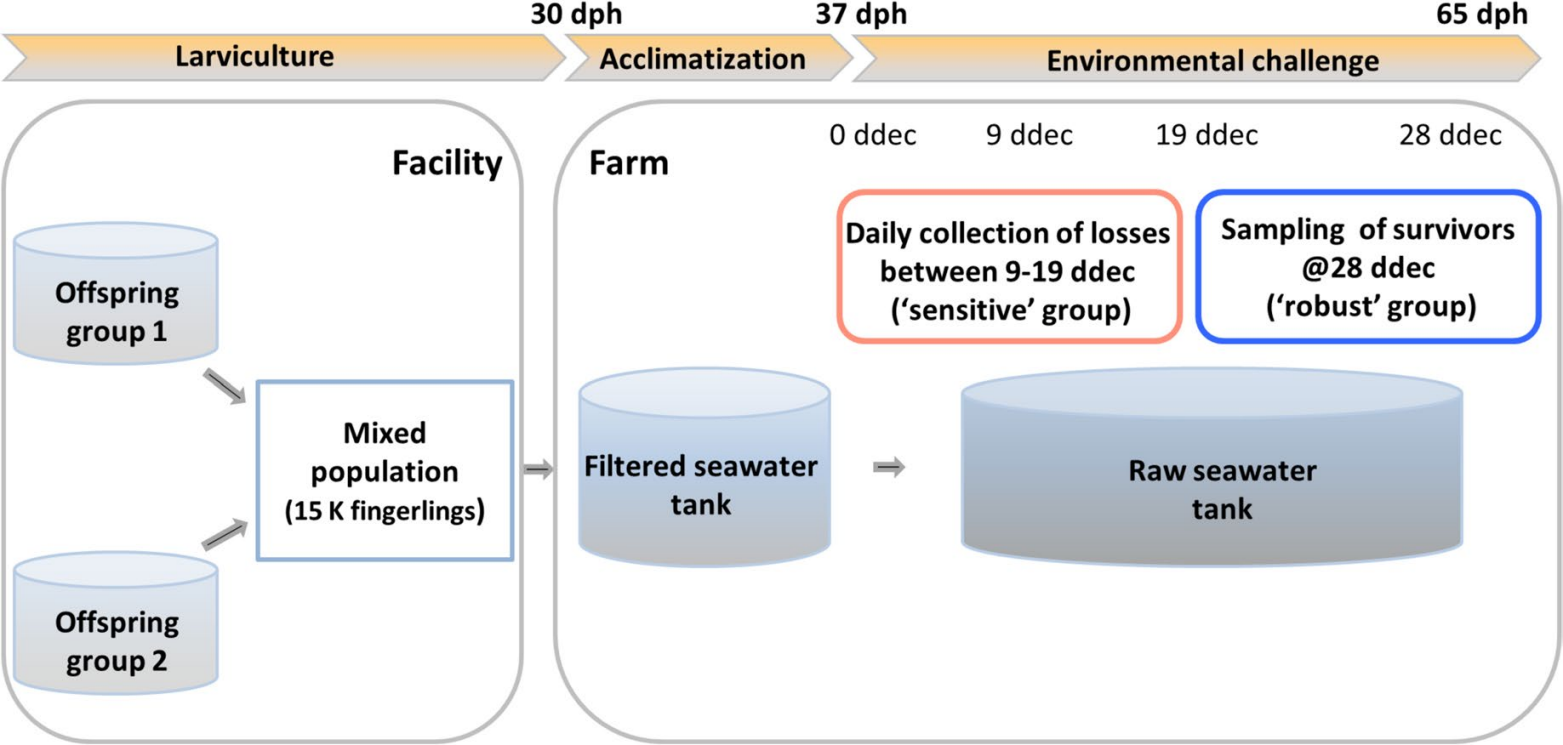
A schematic representation of the complete procedure of the challenge. Two offspring groups were produced by mass-crossing selected brooders using two separate spawning. The two batches were grown separately in clean, sand-filtered seawater up to 30 dph (day post-hatch), when they were mixed, and ca. 15 thousand unvaccinated fingerlings were transferred to a floating fish farm located in the coastal area of Singapore. At the farm, fingerlings were acclimatized for a week in filtered, ozonized and UV-treated seawater, then they were transferred into 2,000-L tanks containing aerated raw seawater (flow-through) at ambient temperature (28–30 °C). The mortalities and moribund individuals were collected, counted and archived on a daily basis. The 500 individuals lost during the earliest days of 9 to 19 ddec (days during environmental challenge) served as ‘susceptible’ or ‘sensitive’ individuals. The 750 survivors without any symptoms of pathogen infection at the end of the whole experiment (28 ddec) were randomly sampled as ‘robust’ individuals

In the current study, ca. 15 thousand fingerlings representing five families were subjected to the environmental challenge at the age of 37 dph (i.e., seven days post-transfer to the farm), and the challenge lasted for 28 days (Fig. 1).

The mortalities have started at around nine days after the initiation of the treatment (‘days during environmental challenge’ or ddec in short). Moribund/dead individuals were collected daily and stored in groups in absolute ethanol at 4 °C according to the number of days from challenge to morbidity/mortality. The first 500 moribund or dead specimen collected during the 9–19 ddec period formed the ‘sensitive’ group for the genotyping. Five of these individuals were examined for signs of potential pathogen infection by histological analysis (Supplementary Fig. S3). The following clinical signs were observed on most of them: thin body, mild erosion of dorsal fin, moderately pale gills, dark red livers, swollen kidneys, moderate reddening of mouth and around the eyes bilaterally. Histology results showed extensive fulminating granulomatous inflammation with hemorrhage in the intestine, with large numbers of ‘big belly’-like bacteria scattered throughout the tissue (enteritis). There were also multifocal areas of intracellular ‘big belly’-like bacteria in the liver, with a moderate infiltrate of lymphocytes (hepatitis). In addition, many Trichodinids located between the primary gill lamellae, and *Vibrio alginolyticus* were also isolated. Survivors at 28 ddec – without any obvious symptoms of pathogen infection – were randomly sampled to form the ‘robust’ group (750 individuals) for genotyping. We chose this timepoint for the termination as the number of mortalities has dropped compared to the previous days. At that point, the ‘robust’ survivors spent 9–19 days longer in the pathogen-infested seawater than their ‘sensitive’ siblings and as opposed to them, the ‘robust’ individuals have not seemed to be affected by the pathogens.

### A high-resolution genetic linkage map containing 3,089 SNPs was constructed

**Fig. 2:**
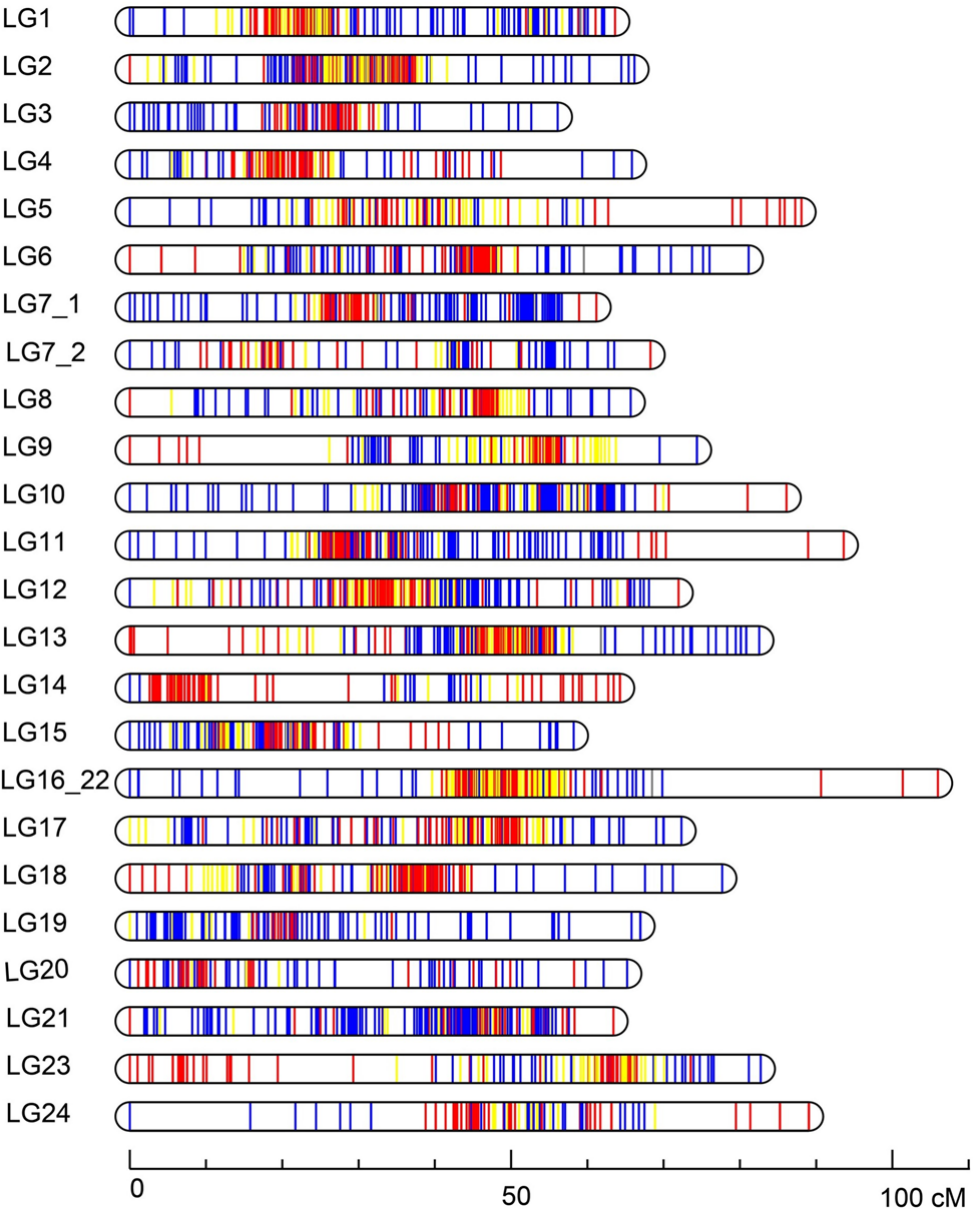
The integrated genetic linkage map and distribution of genetic markers along each linkage group of the Asian seabass. Blue, red and yellow vertical lines in the linkage groups represent maternal heterozygous SNPs, paternal heterozygous SNPs, and SNPs heterozygous in both parents, respectively

The ddRAD libraries were generated from the genomic DNA of two brooders and their 172 offspring of the mapping family and sequenced on an Illumina NextSeq 500 platform. A total of 660.86 million clean reads corresponding to 97.10 Gb sequence were produced from the 174 samples (Supplementary Table S1). Among them, 7.51 million (1.10 Gb) and 7.84 million (1.15 Gb) clean reads were from the female and male brooder, respectively. In addition, 94.85 Gb clean reads with an average of 551.44 Mb per specimen for all the offspring were produced for individual SNP discovery (Supplementary Table S1). A total of 12,330 raw polymorphic SNPs was detected using the STACKS pipeline. After further filtration with more stringent conditions (missing rate < 10%, *P* < 0.01 for segregation ratio Chi-Square χ^2^ test), a consensus map containing 3,089 segregating SNP markers was created. It contained 24 linkage groups (LGs) with total map length of 1,771 cM (Table 1; Fig. 2). The genetic length of individual LGs ranged from 56.12 cM (ASB_LG3) to 105.96 cM (LG16_22), with an average of 73.77 cM. The number of markers for each LG ranged from 70 in LG24 to 205 in LG2 (average: 129). Marker intervals ranged from 0.32 cM (ASB_LG2) to 1.27 cM (ASB_LG24), with an average of 0.57 cM. The recombination rate in comparison to the physical map was from 1.89 cM/Mb for LG15 to 4.91 cM/Mb for LG7-2, with an average of 3.15 cM/Mb (Table 1).

**Table 1:** Summary statistics of the integrated genetic linkage map of Asian seabass

LG_ID	Integrated map					
	**No. of mapped markers**	**Genetic length (cM)**	**Average marker interval (cM)**	**Largest gap (cM)**	**Physical length (Mb)**	**cM/Mb**
**1**	157	63.64	0.41	4.27	25.7	2.48
**2**	205	66.16	0.32	4.18	30.39	2.18
**3**	81	56.12	0.69	6.77	23.5	2.39
**4**	101	65.85	0.65	10.67	25.54	2.58
**5**	90	88.06	0.98	16.3	28.96	3.04
**6**	137	81.15	0.59	5.89	27.92	2.91
**7_1**	145	61.19	0.42	4.69	23.26	2.63
**7_2**	86	68.27	0.79	4.74	13.91	4.91
**8**	112	65.68	0.59	5.47	25.92	2.53
**9**	100	74.37	0.74	17.04	22.99	3.23
**10**	176	86.11	0.49	10.34	27.94	3.08
**11**	127	93.60	0.74	18.66	23.29	4.02
**12**	179	71.96	0.4	3.86	27.84	2.58
**13**	203	82.53	0.41	8.06	27.24	3.03
**14**	86	64.28	0.75	9.88	14.07	4.57
**15**	138	58.22	0.42	4.99	30.78	1.89
**16_22**	156	105.97	0.68	20.79	25.85	4.10
**17**	128	72.34	0.57	4.30	27.67	2.61
**18**	125	77.68	0.62	6.45	19.19	4.05
**19**	105	66.95	0.64	8.19	24.52	2.73
**20**	84	65.22	0.78	7.55	23.75	2.75
**21**	195	63.41	0.33	5.09	28.68	2.21
**23**	103	82.72	0.8	9.92	18.17	4.55
**24**	70	89.04	1.27	15.81	19.81	4.49
**Total**	3,089	1,771	/	/	586.92	/
**Average**	129	73.77	0.57	8.91	23.48	3.15

### Identification of a major QTL and 36 potential candidate genes associated with increased robustness in a complex, pathogen-infected marine environment

**Fig. 3:**
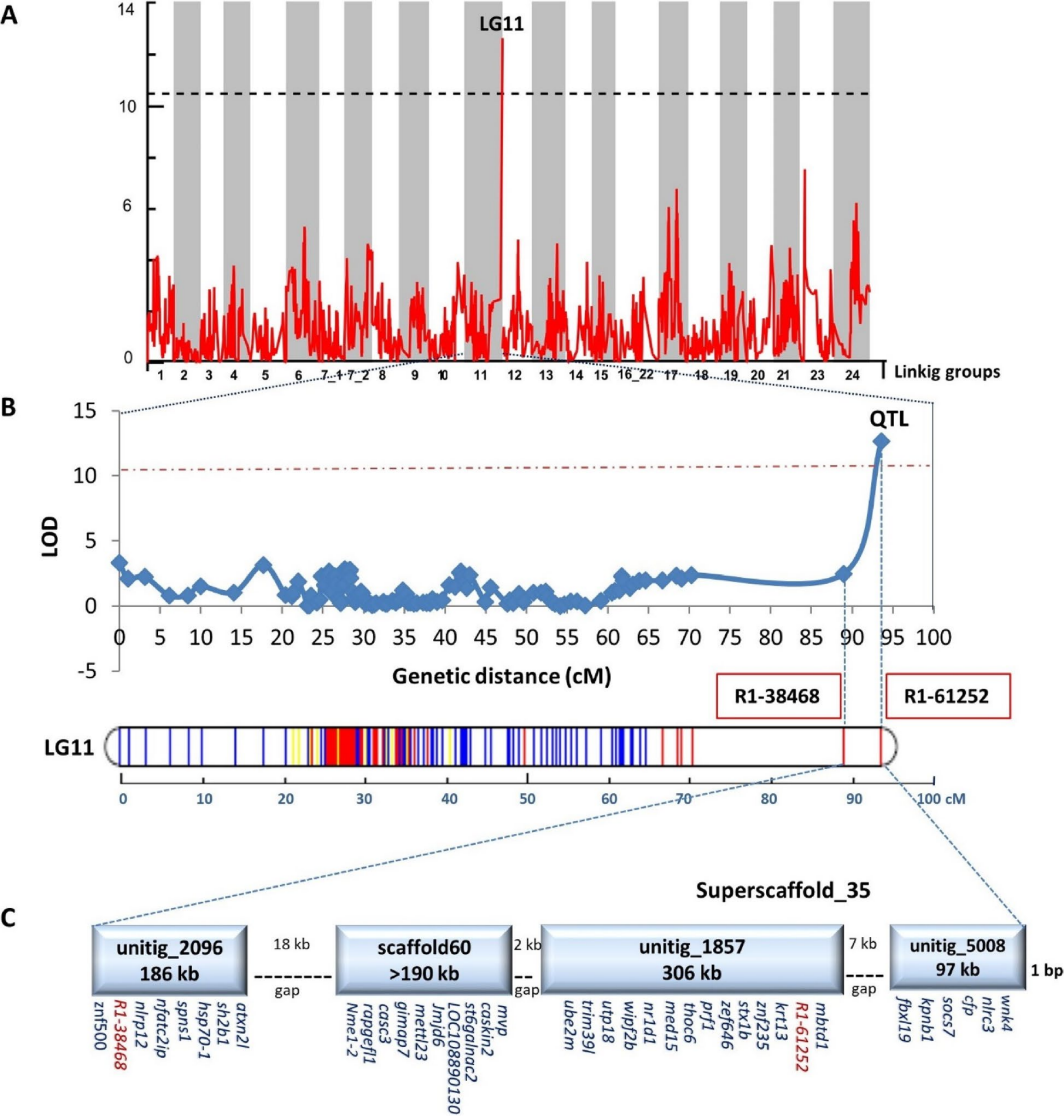
Genome-wide scan identified a QTL significantly associated with increased robustness on ASBB_LG11. **A** Genetic location of QTLs associated with increased robustness along the Asian seabass genome. Black horizontal line represents the genome-wide logarithm of odds (LOD) significance threshold of 10.5. X- and Y-axes correspond to the linkage groups and the LOD value, respectively. **B** Localization of the major QTL associated with increased robustness on ASB_LG11. Map positions and LOD scores are based on a single interval mapping QTL analysis using the software MAPQTL6. The 95% genome-wide LOD significance threshold value was ca. 10.5 (dashed constant line). The QTL had a LOD peak of 12.6 with an interval of 4.7 cM region. Two SNPs (R1-38468 and R1-61252) were identified within the interval. R1-61252 was located at the peak position of 93.6 cM. **C** The genomic region of mapped QTL and putative causative candidate genes on ASB_LG11. The QTL was located on Superscaffold_35 (LG11) which comprised of four sequences in the following order: unitig_5008|quiver, unitig_1857|quiver, scaffold_60 and unitig_2096|quiver, scaffold_60, unitig_1857|quiver and unitig_5008|quiver. The superscaffold spanned an over 800 kb genomic region, including estimated gaps of 27 kb. Gene symbols of 36 potential causative genes and their distribution on Superscaffold_35, as well as the two SNP markers are indicated

Interval mapping identified a major QTL (PVE of 80.1%) with genome-wide significance for increased robustness on ASB_LG11 (Fig. 3A), with an interval of 4.7 cM (88.9 cM to 93.6 cM). This region was flanked by two SNPs; the first, called R1-38,468, was located at 88.9 cM and the second, called R1-61,252, was located at the peak position (93.6 cM; Fig. 3B). By mapping the flanking sequences of these two SNPs onto the assembled genome of seabass [35], their corresponding sequences of unitig_1857|quiver (307 kb) and unitig_2096|quiver (187 kb) were identified, respectively. Both unitigs were located within Superscaffold_35, which spanned a genomic region over 800 kb long, including estimated gaps of 27 kb size, and it was located at the end of ASB_LG11 (Fig. 3B). Superscaffold_35 was comprised of four sequences in the following order: unitig_2096|quiver (186 kb), scaffold_60 (> 190 kb), unitig_1857|quiver (306 kb) and unitig_5008|quiver (97 kb) (Fig. 3C).

A search through the seabass genome annotation files identified a total of 36 genes within the above genomic region containing the QTL (Table 2; Fig. 3C). Based on literature searches, BLAST2GO function annotations, KEGG pathway analysis as well as searches in the GeneCards and OMIM databases, the protein products of over two-third (25/36; 69.4%) of these genes seem to possess immune- or disease-related functions (Table 2).

**Table 2:** Thirty-six potential causative genes located within the QTL for increased robustness in Asian seabass

Gene symbol	Gene name	Location	Gene ID	Immune/disease- related function^a^	Detailed function(s) based on GO, GeneCards, KEGG Pathway, KEGG Disease & OMIM databases
*atxn2l*	Ataxin-2-like protein	**@unitig_2096**	LOC108892192	Yes	Regulator of stress granules; gastric cancer
*casc3*	Casc3 exon junction complex subunit	**scaffold_60**	LOC108882395	Yes	mRNA surveillance pathway; inhibits breast cancer progression and metastasis
*caskin2*	Caskin-2 like protein	**scaffold_60**	LOC108882385		Dendritic spine morphology and memory formation
*cfp*	Complement Factor Properdin	**@unitig_5008**	LOC108873264	Yes	Herpes simplex virus 1 infection
*fbxl19*	F-box/LRR-repeat protein 19	**@unitig_5008**	LOC108873267	Yes	Innate Immune System; Class I MHC mediated antigen processing and presentation
*gimap7*	GTPase IMAP family member 7-like	**scaffold_60**	LOC108882392	Yes	Lymphocyte development; immune system homeostasis
*hsp70-1*	Heat shock 70 kDa protein 1	**@unitig_2096**	LOC108892190	Yes	Influenza A; Legionellosis; Antigen processing and presentation; Measles; Epstein-Barr virus infection
*jmjd6*	Jumonji domain containing 6	**scaffold_60**	LOC108882389	Yes	T cell differentiation in thymus; Chromatin organization
*kpnb1*	Karyopherin (importin) beta 1	**@unitig_5008**	LOC108873261		RNA transport
*krt13*	Keratin, type I cytoskeletal 13	**@unitig_1857**	krt13	Yes	Congenital malformations
*LOC108890130*	Tsetse EP-like protein	**scaffold_60**	LOC108882390	Yes	Protects fly midgut from trypanosome establishment
*mbtd1*	MBT domain-containing protein 1	**@unitig_1857**	LOC108891038	Yes	Tumorigenesis
*med15*	Mediator complex subunit 15	**@unitig_1857**	LOC108891028		Developmental biology; PPARA activates gene expression
*mettl23*	Methyltransferase-ike 23	**scaffold_60**	LOC108882391	Yes	Autosomal recessive mental retardation; Mental and behavioural disorders
*mvp*	Major vault protein	**scaffold_60**	LOC108882383	Yes	Innate Immune System; tumorigenesis; drug resistance
*nfatc2ip*	Nuclear factor of activated T-cells 2 interacting protein	**@unitig_2096**	LOC108892221	Yes	RNA transport; Fluid shear stress and atherosclerosis
*nlrc3*	NACHT, LRR and PYD domains-containing protein 3	**@unitig_5008**	nlrc3	Yes	NOD-like receptor signaling pathway; influenza virus infection
*nlrp12*	NACHT, LRR and PYD domains-containing protein 12	**@unitig_2096**	LOC108892182	Yes	NOD-like receptor signaling pathway; Familial cold autoinflammatory syndrome 2; MHC induction
*nme1-2*	Nucleoside diphosphate kinase, A2-like	**scaffold_60**	LOC108882397	Yes	Drug metabolism; activation of CD4 T cells
*nr1d1*	Nuclear receptor subfamily 1, group D, member 1	**@unitig_1857**	LOC108891029		Circadian rhythm
*prf1*	Perforin 1.3	**@unitig_1857**	LOC108891017	Yes	Natural killer cell mediated cytotoxicity; Type I diabetes mellitus; Apoptosis; Autoimmune thyroid disease; Allograft rejection; Graft-versus-host disease; Viral myocarditis
*rapgefl1*	Rap guanine nucleotide exchange factor (GEF)-like 1	**scaffold_60**	LOC108882396		Rap1 signaling pathway; Ras signaling pathway
*sh2b1*	SH2B adapter protein 1-like	**@unitig_2096**	LOC108892191		Neurotrophin signaling pathway
*socs7*	Suppressor of cytokine signaling 7	**@unitig_5008**	LOC108873260		Jak-STAT signaling pathway
*spns1*	Sphingolipid transporter 1 (putative)	**@unitig_2096**	LOC108892183	Yes	MAPK signaling pathway; induction of necrotic cell death via autophagy
*st6galnac2*	ST6 (alpha-N-acetyl-neuraminyl-2,3-beta-galactosyl-1,3)-N-acetylgalactosaminide alpha-2,6-sialyltransferase 2	**scaffold_60**	LOC108882387		Synthesis of substrates in N-glycan biosythesis; O-linked glycosylation of mucins
*stx1b*	Syntaxin-1B	**@unitig_1857**	LOC108891022	Yes	SNARE interactions in vesicular transport; Febrile seizures and nervous system diseases
*thoc6*	THO complex 6	**@unitig_1857**	LOC108891025	Yes	Congenital malformations
*trim39l*	E3 ubiquitin-protein ligase TRIM39-like	**@unitig_1857**	LOC108891033	Yes	Innate Immune System; Class I MHC mediated antigen processing and presentation
*ube2m*	Ubiquitin conjugating enzyme E2M	**@unitig_1857**	LOC108891035		Ubiquitin mediated proteolysis
*utp18*	UTP18 small subunit processome component	**@unitig_1857**	LOC108891031		Ribosome biogenesis in eukaryotes
*wipf2b*	WAS/WASL interacting protein family, member 2b	**@unitig_1857**	LOC108891032	Yes	Yersinia infection; Pathogenic Escherichia coli infection; Endocytosis
*wnk4*	Serine/threonine-protein kinase WNK4	**@unitig_5008**	LOC108890345	Yes	Hyperkalemic distal renal tubular acidosis (RTA type 4); Urinary system diseases; Kidney diseases
*znf235*	Zinc finger protein 235	**@unitig_1857**	LOC108891019	Yes	Herpes simplex virus 1 infection
*znf500*	Zinc finger protein 500-like	**@unitig_2096**	LOC108892188		Generic Transcription Pathway
*znf646*	Zinc finger protein 646	**@unitig_1857**	LOC108891016	Yes	TLR-triggered innate immune response

^a^Genes not labeled with 'Yes' might still have immune- or disease-related function

### Validation of a SNP significantly associated with increased robustness in a mass-cross population

**Fig. 4:**
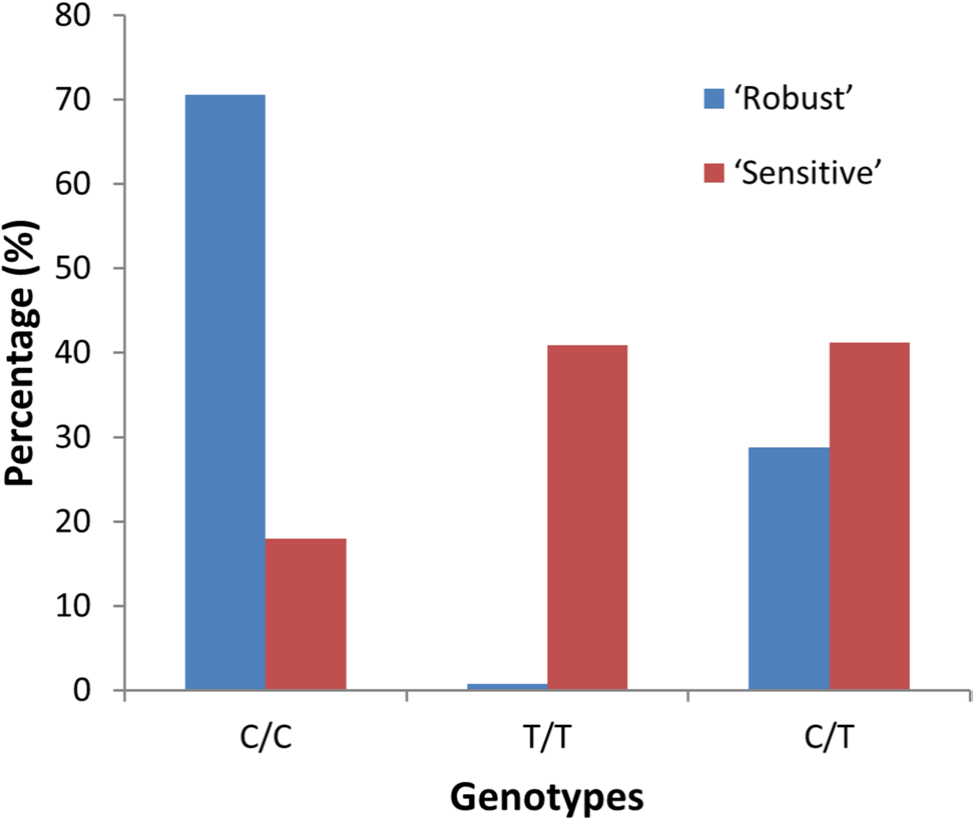
The association of SNP marker R1-61252 to robustness was validated in a different stock of Asian seabass. We found significant differences in genotype frequencies at this SNP between the ‘sensitive’ (red) and ‘robust’ (blue) groups as examined (*p* < 0.01, Chi-square test). The C/C genotype was nearly four times more frequent in the ‘robust’ group (203/288 individuals) than in the ‘sensitive’ one (46/255); whereas T/T was much less common in the ‘robust’ group (2/288) than in the ‘sensitive’ one (104/255; over 59-fold difference). The C/T genotype was somewhat more common in the ‘sensitive’ group ( (105/255) than in ‘robust’ one (83/288; a 1.4-fold difference)

The R1-61,252 SNP marker, that was located at peak position within the QTL region, was further analyzed in a different mass-cross population consisting of 543 offspring individuals (255 ‘sensitive’ and 288 ‘robust’ ones). We found significant differences in genotype frequencies at this SNP between the ‘sensitive’ and ‘robust’ groups as examined (*p* < 0.01, Chi-square test). The C/C genotype was observed far more frequently in the ‘robust’ group (203/288 individuals) than in the ‘sensitive’ group (46/255 individuals; a nearly four-fold difference); whereas the T/T genotype was much more frequent in ‘sensitive’ individuals (104/255) than in the ‘robust’ ones (2/288; over 59-fold difference; Fig. 4). The C/T genotype was somewhat more common in the ‘sensitive’ group (105/255) than in ‘robust’ one (83/288; a 1.4-fold difference). Allele frequencies for the ‘C’ and ‘T’ alleles in the ‘robust’ group were 84.9% and 15.1%, respectively; whereas the corresponding values in the ‘sensitive’ group were 38.6% and 61.4%, respectively. This indicated that the ‘C’ allele was associated with robustness, while the ‘T’ allele with susceptibility.

### The ddRAD map allowed for the detection of five mis-assemblies in the seabass genome

Comparison of the consensus RAD map to the seabass genome assembly labelled as v2 (contigs/scaffolds assembly) [35] showed that all, but one, of 3,089 SNPs successfully mapped to 596 out of 3,807 contigs/scaffolds. Among the mapped sequences, 189 (31.7%) contained a single SNP each, while the remaining of 407 (68.3%) contained two or more SNPs. In 19 out of these latter sequences, the contained SNPs unexpectedly mapped to two or three separate linkage groups, indicating putative mis-assemblies within these sequences (Supplementary Table S4). When syntenic relationships of SNPs in each linkage group with their corresponding chromosome-level genome assembly v3 [35] were checked, two SNPs showed no alignment, whereas 29 SNPs corresponding to six scaffolds/unitigs showed mismatches between the specific LGs and their corresponding chromosomes (Supplementary Table S5). Among these 25 (i.e., 19 + 6) putative mis-assemblies, five were found to be present in both the v2 and v3 genome assemblies. Therefore, total of 20 mis-assembled sequences, including four unitigs and 16 scaffolds, were discovered in the end (Supplementary Table S6). Fifteen out of these mis-assemblies had already been corrected in v3 by taking advantages of the shared syntenic analysis. Thus, five putative mis-assemblies, namely unitig_2144 on ASB_LG9, unitig_4383 and unitig_4955 on ASB_LG8, unitig_4480 on ASB_LG20, and scaffold_18 on chromosome ASB_LG15, were detected in the current published seabass reference genome v3 (Table 3; Supplementary Table S6).

**Table 3:** Five mis-assemblies in the Asian seabass genome identified by RADSeq map

ID of miss-assembled unitigs (scaffolds)	unitig_2144	unitig_4383	unitig_4480	unitig_4955	scaffold_18
Original length of mis-assembled sequences (bp)	144,568	2,107,682	1,225,529	2,202,706	14,175,190
SNPs mapped	4	2	7	4	87
Mapped linkage group ID (No. of mapped markers)	LG14 (4)	LG12 (2)	LG12 (3)	LG8 (3)	LG10 (12)
			LG20 (4)	LG23 (1)	LG14 (16) LG15 (59)
Mapped Chromosome ID (No. of mapped markers)	ASB_LG9 (4)	ASB_LG8 (2)	ASB_ LG20 (7)	ASB_LG8 (4)	ASB_LG10 (12) ASB_LG15 (75)
Mis-assembled fragment position on unitigs (scaffolds) (bp)	5,976 -129,886	1,720,878—2,040,779	669,670—1,186,545	1,980,792—2,054,320	11,650,152—14,151,079
Mis-assembled fragment size unitigs (scaffolds) (bp)	123,919	319,901	516,875	73,528	2,500,927
ID of mis-assembled chromosomes	ASB_LG9	ASB_LG8	ASB_LG20	ASB_LG8	ASB_LG15
Mis-assembled region on chromosomes (bp)	20,764,709- 20,888,619	9,863,474—10,183,375	1,868,914—2,385,789	14,315,729—14,389,257	18,305,472—20,806,399
Newly assigned chromosome for mis-assembled fragments	ASB_LG14	ASB_LG12	ASB_LG12	ASB_LG23	ASB_LG14

**Fig. 5:**
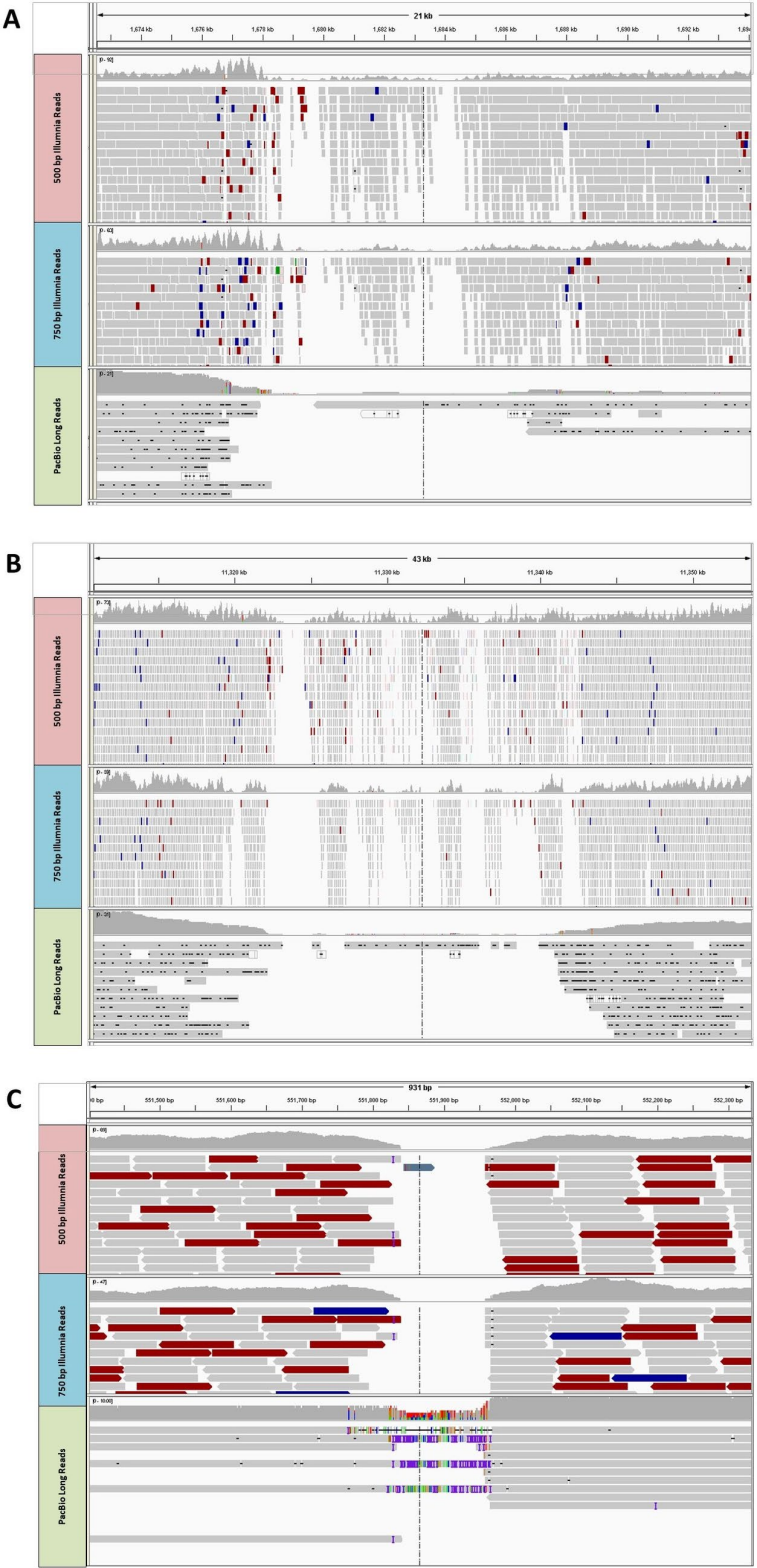
Three examples of the mis-assembled sequences detected by the ddRAD map. **A** The first breakpoint (1,676–1,688 kb) within scaffold_18 was detected earlier and confirmed by ddRAD map. This has also been found earlier by synteny analysis, but not by optical mapping. **B** The second breakpoint (11,315–11,345 kb) was newly detected by the ddRAD map. Both breakpoints were revealed by Illumina short reads from both 500 and 750 insert size libraries as well as long PacBio reads by having low or zero physical coverage of all three libraries. **C** A breakpoint identified within unitig_4480 by the ddRAD map. The breakpoint was revealed by Illumina short reads from both 500 and 750 short insert size libraries by having no physical coverage as well as high density of repeats located within those few PacBio-based long reads that were mapped there

To further validate these five putative mis-assemblies, their locations/regions were first narrowed by utilizing the ddRAD map. Then their regions were inspected through REAPR [36] by alignments of Illumina short paired-end reads from two short insert libraries (500 bp and 750 bp) and 90X PacBio long reads. The alignment results showed that the putative mis-assembled regions among the five unitigs and/or scaffolds had either low physical coverage or a complete lack of coverage of the PacBio read alignments and two short insert Illumina libraries (e.g. scaffold_18) (Fig. 5A-B), or high density of repeats locating among those few mapped PacBio long reads (e.g., unitig_4480) (Fig. 5C). Among the above five mis-assemblies, the most interesting finding was Scaffold_18 (14,175 Mb), as in the published reference genome [35], it was split into two fragments on ASB_LG10 and ASB_LG15 by taking the advantage of syntenic relationship analysis [optical mapping didn’t detect any mis-assembled region(s) among this scaffold] (Fig. 5A). However, an additional new breakpoint (Fig. 5B) was further detected in this scaffold within ASB_LG15 by utilizing the current ddRAD map, the mis-assembled region in Scaffold_18 was from 11,650,152 to 14,151,079 bp, corresponding to the mis-assembled genomic region of 18,305,472 to 20,806,399 bp on ASB_18. Therefore, a large fragment with nearly 2.5 Mb size needs to be reassigned to ASB_LG14 (Table 3).

In summary, a total of two breakpoints were detected within Scaffold_18 which need to be broken into three chromosomes of ASB_LG10, ASB_LG14 and ASB_LG15 (Table 3; Fig. 5A-B). For the other four mis-assemblies, one wrongly assigned fragment from each of them needs to be relocated to their own correct chromosome (Table 3). Additionally, by utilizing the ddRAD map, 100 previously unplaced contigs/scaffolds with a total length of 13.1 Mb can now be placed onto a total of 22 out of 24 chromosomes for potential further improvement of the published seabass reference genome v3 (Supplementary Table S7).

## Discussion

### Identification of a major QTL responsible for increased robustness of seabass in a pathogen-containing marine environment

Disease outbreaks cause major problems for farming of seabass in Southeast Asia. The survival of fry and fingerlings can be severely influenced by interactions among the host, pathogen and environmental factors, resulting in major economic losses at fish farms. In our study, we aimed to identify seabass individuals with increased disease tolerance by performing challenges on fingerlings generated by mass crosses under typical farm conditions that are directly relevant for commercial production. Histological studies of seabass individuals infected under such conditions showed the presence of various pathogens, including ‘big belly’ bacteria, *Vibrio alginolyticus* and Trichodinids. In addition to these pathogens, environmental effects and exposure to stressors could also reduce the immunity of fish and cause mass mortality during the challenge.

Until now, no QTLs responsible for increased robustness in seabass have been reported. Previously, several QTLs with relatively small effects (2.2 to 11.0%) on increased resistance against viral nervous necrosis [23, 24, 37] and four QTLs with PVE ranging of 7.5 to 15.6% for Singapore grouper iridovirus [26] were identified for the species based on controlled laboratory experiments on defined families. Our current study identified a major QTL associated with increased robustness (under pathogen-rich natural marine environment) of seabass with PVE of 80.1% on chromosome ASB_LG11. The location of our QTL showed no overlap with those described earlier, as none of the 17 QTLs found in individuals subjected to single pathogen challenges was located at ASB_LG11 [23, 37]. Similarly, to our finding, major QTLs with large effect on resistance to viral, bacterial or parasitic disease(s) have also been reported in other fish species using QTL mapping or GWAS analyses. For example, a QTL explains 50–86% of PVE to whirling disease resistance across families was detected in rainbow trout [38]. A major QTL with a PVE of 29.6% for resistance against Benedenia disease was identified in Japanese amberjack or yellowtail [39]. One of the most successful examples of QTL analyses applied to selective breeding is the case of resistance to IPNV in Atlantic salmon, where a major QTL was first detected on post-smolts and explained up to 79% of the phenotypic variance in four segregating families [20]. Following this finding, two major QTLs associated with resistance to IPNV were then further detected with the PVE of 50.9% [40] and 29% [21], respectively, and demonstrated as a successful means of controlling the disease. In addition, a major QTL contributing 50% of PVE for resistance against the viral lymphocystis disease in Japanese flounder has also been successfully mapped and used in marker-assistant breeding [22, 41]. The discovery of a major QTL responsible for increased robustness in this study is expected to move the field closer to a mechanistic understanding the increased tolerance of seabass – and possibly other cultured marine tropical species – against adverse effects exerted by a complex natural marine environment consisting multiple pathogens.

QTL analyses provide primary information on genetic architecture of disease resistance. However, prior to utilizing the markers in MAS or GS, it is essential to examine them in other families or populations. Here, a study of the R1-61,252 SNP marker located at the peak of QTL revealed significant association with increased robustness in a multifamily population—generated by mass-crossing a group of brooders—which was also challenged under natural environment. The fact that that the two homozygous marker genotypes (i.e., C/C and T/T) showed distinctly different distribution between the ‘robust’ and ‘sensitive’ groups indicates that this SNP could be a promising candidate marker for selecting robust broodstocks of seabass after further validating its performance in additional stocks and populations.

### The majority of potential candidate genes within the QTL have known functions related to immunity, stress responses and human disease

A detailed examination of potential candidate genes was conducted to provide insights into genes and pathways associated with increased robustness of seabass. Interestingly, 25 of the 36 identified genes within the QTL region have been shown to play potential roles in immunity, stress responses, and human disease. Here, we discuss the information available about the function of four of them in detail.

Both *nlrc3* and *nlrp12* are NOD-like receptors, that belong to the NB-LRR-related (NLR) gene family [42]. Several studies on fish species, including Japanese flounder [43] and channel catfish [44], indicated the function of *nlrc3* in the activation of innate immune responses following bacterial and virus infection. In seabass, a previous study that analyzed the infection process of V. *alginolyticus* suggested that *nlrc3* could be a pivotal cytosolic innate immune receptor against wide array of pathogens (Gram-negative bacteria and viruses) in diverse environmental conditions [45]. A previous GWAS study on channel catfish subjected to *Edwardsiella ictaluri* infection identified *nlrp12* within a significant QTL region, which contains important early mediators of innate mucosal responses [44]. All these data suggest that both *nlrc3* and *nlrp12* may play an important role in the activation of innate immune responses following infection in seabass, but further functional studies will be required to determine their exact roles.

The stress-induced heat shock protein 70 (Hsp70) has been shown to enhance the tolerance of aquatic organisms against diseases [46]. Studies on fish and shrimp have suggested that a non-lethal heat shock can promote the synthesis of Hsp70 and enhance the resistance of larvae against pathogen infection [47–50]. In seabass, *hsp70* has been previously identified as one of the top differentially expressed genes in response to NNV infection [51]. Complement factor properdin (*cfp*), encodes a plasma glycoprotein that regulates the alternative complement pathway of the innate immune system [52]. Its expression profile in infected channel catfish has also been analyzed. A pathogen-specific pattern of regulation was found, indicating that *cfp* may play a complex role in the host immune responses to bacterial pathogens [53]. In seabass, *cfp* was also identified previously as one of the top significantly DEGs upon challenging with LPS to identify pathways that are activated following bacterial infection [54]. In this study, the identification of *cfp* suggesting that it might play a crucial role in the disease defense response processes of seabass.

Although a more limited amount of data is available regarding the roles of the remaining genes in other fish species, several of them are closely associated with different types of human disease. These putative candidate genes and the pathways they are involved with might provide clues for selecting more robust seabass lines less prone to severe infection by pathogenic microorganisms present in the complex aquatic environment.

### The ddRAD map provided a chromosomal framework for validating and improving the current seabass reference genome assembly

The seabass reference genome (v3) includes 24 chromosomal scaffolds (782 unitigs and 85 scaffolds) and 2,940 unplaced sequences, with a total number of 3,807 sequences [35]. Although the quality of the assembly exceeded that of most other fish species at the time of its publication, the 22,184 of annotated genes was relatively low compared to the average gene number in other teleosts. This could be caused by genome assembly errors and incompleteness, as many coding genes are located in large blocks of highly differentiated and sometimes repetitive sequence which are difficult to assemble. High density linkage maps have been utilized for validating genome assemblies of several aquaculture species. For instance, a RAD map consisting of 1,085 SNPs revealed widespread errors in oyster genome scaffold assembly [31], with total 100 out of 618 (16.2%) scaffolds which containing two or more mapped SNPs mapped to different LGs. In a study performed for improving Nile tilapia genome assembly through introducing 44X coverage of PacBio reads, 21 putative interchromosomal mis-assemblies were identified by RAD map [32]. In channel catfish, five mis-assembled regions were found based on the differences of the marker orders between the linkage map and the reference genome sequences [33]. In our study, we set out to identify potential mis-assemblies within seabass reference genome by utilizing the ddRAD map constructed here. At first, 20 out of 3,807 (0.5%) mis-assembled sequences were discovered, a ratio far lower than those of the above studies. Further analysis showed that only five (0.1%) mis-assembled sequences could be detected in the current genome, as 15 out of the 20 mis-assemblies identified here had already been corrected in v3. Such a low ratio of mis-assemblies pointed to a high accuracy on connectivity and contiguity of the current seabass reference genome assembly.

In addition, this ddRAD map also validated 20 out of 28 mis-assembles, which were identified previously in the v3 assembly by utilizing syntenic analysis before they were assigned onto chromosomes to create the chromosomal level seabass genome [35]. However, a single breakpoint only was detected earlier for scaffold_18 by syntenic analysis (optical mapping did not detect any errors within it), while a second breakpoint was discovered by utilizing the ddRAD map constructed in this study. The addition of this second breakpoint split this scaffold among three chromosomes (ASB_LG10, ASB_LG14 and ASB_LG15) instead of two (ASB_LG10 and ASB_LG15). Moreover, four additional mis-assembles on chromosomes ASB_LG8, ASB_LG9, ASB_LG15 and ASB_LG20 were consistent with an earlier study, except for ASB_LG2 which was not detected in this study [55]. However, here we pointed out the position of the mis-assembled fragments on the chromosomes, and indicated the corresponding chromosomes these fragments needed to be reassigned to (Table 3). All in all, these data suggested that the high-density linkage map reported in this study is a useful tool for validating and further improving the current seabass genome assembly.

## Conclusions

A high-resolution linkage map was constructed based on 3,089 SNPs for Asian seabass. A single major QTL (PVE of 80.1%) located on ASB_LG11 and responsible for increased robustness in complex pathogen-infected marine environment was identified. The majority of the 36 putative candidate genes within the QTL have known function related to immunity, human disease and stress responses. Moreover, an SNP located at the peak of QTL was revealed to be significantly associated with increased robustness trait in a multifamily population. All these outcomes have laid the foundation for MAS, GWAS and GS of seabass lines with increased robustness when kept at complex natural environment. Such lines are expected to be useful for farmers who are aiming to breed more robust seabass that will hopefully lead to reduced losses during production. Additionally, five mis-assembled sequences corresponding to four chromosomes were detected and further confirmed, revealing the ddRAD map as an important tool for improving the current Asian seabass genome assembly.

## Materials and methods

### Farm-based challenges

Our research team conducted 14 challenges at Singapore-based floating fish farms utilizing raw sea water between 2013 and 2016. In general, brooders were spawned in a mass cross, then their offsprings were grown in sand-filtered sea water for about a month. Next, they were transferred to a local floating fish farm where following a week-long acclimation period they were grown in raw sea water. Mortalities were collected regularly and analyzed during the next 1–2 months period.

In the current study, the offspring groups were produced by mass-crossing selected F1 and F2 brooders through hormone-induced spawning using two spawning tanks containing a total of nine female and six male brooders at the Marine Aquaculture Center of the Singapore Food Agency (see Fig. 1 for the outline of the experiment). Fin clips from each potential parent were collected at the time of spawning. At 3 dph (day post-hatch), around 500 larvae from the two tanks were randomly collected for genotyping to determine the level of contribution of families to the offspring by using a multiplex genotyping set consisting of 10 primer pairs flanking polymorphic microsatellites [56]. A total of five families were identified to contribute to this population of offsprings. At 30 dph, an estimated number of 15,000 unvaccinated fingerlings with an average body weight of 0.5 ± 0.02 g were mixed equally from the two tanks and transferred to a floating fish farm located in the coastal area of Singapore. After one-week acclimatization in a filtered seawater tank (the raw seawater was filtered to 5 µm followed by ozone and UV sterilization), the offspring were split into three groups and transferred into three 2,000-L tanks with aerated flow-through raw seawater and saturated oxygen at ambient temperature (28–30 °C). The fish were fed twice a day with a commercial diet (Otohime EP1) during the whole experiment. The fish were subjected to the field environment challenge at 37 dph (i.e., 7 days post transfer to the farm), and the collection of mortalities started at this point. The loss of fingerlings started to intensify at nine days during environmental challenge (ddec) and continued throughout the 28 days-long treatment period. Five hundred mortalities and moribund individuals (the latter humanely euthanized by using AQUI-S™ (40 mg/L) immersion baths) collected between 9 and 19 ddec served as ‘sensitive’ individuals. The estimated number of 4,500 survivors without any symptoms of pathogen infection at the end of the whole experiment (28 ddec) were randomly sampled to yield the 750 ‘robust’ individuals (see Fig. 1 for schematic representation of the overall setup). A caudal fin sample was collected from each mortality and a set of survivors, then stored in absolute ethanol at -20 °C prior to DNA extraction and genetic analysis. In addition, four euthanized moribund fingerlings were dissected to collect tissues (major organs such as liver, kidney, spleen, intestine and gill etc*.*) that were fixed in 10% phosphate-buffered formalin. Formalin fixed tissues were sent to Singapore Food Agency Veterinary Histology Laboratory for histoprocessing into haematoxylin and eosin (H&E) slides.

### Mapping family, ddRAD library construction and sequencing

Genomic DNA was isolated from the fins of the 15 brooders, and that of 500 ‘sensitive’ individuals as well as 750 ‘robust’ ones by using the high salt precipitation method [57]. Although the potential brooders were known before challenge experiment, but offspring were mixed for communal culture. Therefore, parentage assignment was undertaken by genotyping brooders, ‘sensitive’ and ‘robust’ offspring individuals using the above multiplex PCR set [56]. A total of five families were shown to contribute to these selected sets of individuals; one full-sib family with 81 ‘sensitive’ and 91 ‘robust’ individuals, was chosen as a panel for constructing a linkage map and QTL mapping.

Genomic DNA samples from the selected mapping family were quantified using Qubit® assays (Life Technologies, USA), and their concentrations were adjusted to 50 ng/µl using TE buffer. RAD libraries were constructed using the double digest RADseq (or ddRADseq) approach [58]. In brief, 500 ng of DNA from each sample was double-digested with 20 units of EcoRI-HF and NIaIII (New England Biolabs, USA) for 30 min at 37 °C. Digested DNA fragments were examined by electrophoresis and then ligated with barcoded adaptors of P1 that contained a matching sticky-end and a MID (a short sequence that will uniquely identify the individuals) and P2. After cleaning up with AMPure® XP beads (Beckman Coulter, USA), the ligation products were pooled for size selection with Pippin Prep (Sage Science, USA). The fragments from the 300 bp to 500 bp size range were selected and amplified using Phusion® High-Fidelity DNA Polymerase (New England Biolabs, USA). Finally, the PCR products were cleaned up with AMPure® XP beads and quantified with KAPA Library Quantification Kits (Kapa Biosystems, USA) for paired-end sequencing (2X150 bp) on NextSeq 500 (Illumina, USA).

### SNP discovery and genotyping

First, the Illumina short reads lacking sample-specific molecular identifiers (MIDs) and expected restriction enzyme motifs were filtered out. Then, reads were further filtered on the basis of quality score using Trimmomatic v0.32 [59] in three steps: (1) removing adapters and index; (2) removing reads with average quality less than 20 or containing more than 10% Ns; and (3) scanning the reads with a 4-bp sliding window, removing the read when the average Phred quality per base was below 10. The STACKS pipeline [60] was used to assemble loci de novo from the sequencing data for SNP calling. USTACKS, CSTACKS, SSTACKS, and GENOTYPE programs were used to create libraries of loci, i.e., one for each individual and one for all loci shared among individuals. The detailed parameters are as follows: USTACKS: -t gzfastq -i -m 3 -M 3 -p 15 -d -r –f –o; CSTACKS: -b 1:M 3 -p 15 -d –r; SSTACKS: -b 1 –c –p 15; GENOTYPE: -b 1 –P -r 1 -c -s -t CP. Indels were removed and biallelic sites with genotypes having a minimum genotyping quality of 30 and a minimum of five reads per site per individual were kept only. In addition, the sites genotyped for less than 90% of individuals were excluded as well.

### Genetic map construction and QTL mapping

For linkage analysis, SNPs were first tested against the expected segregation ratio. To construct a genetic map, markers showing significant segregation distortion (*p* < 0.01, Chi-Square test) were removed. The remaining SNPs were then used to construct the genetic map using JoinMap 4.1 [61]. Linkage group assignments were made under the logarithm of odds (LOD) score limit of 7.0. The regression mapping algorithm and Kosambi’s mapping function were used for map construction using default parameters. The resulting linkage maps were drawn using a custom Perl script (https://github.com/Niuyongchao/Fish_linkage_map).

QTLs were identified using MapQTL 6.0 [62] with interval mapping. Automatic cofactor selection (backward elimination, *p* < 0.05) was used for the detection of significantly associated markers as cofactors. The significance thresholds for LOD scores were determined by 1000 permutations. QTL with a LOD score more than the corresponding chromosome-wide LOD threshold at the 0.05 level was considered as significant. The location of each QTL was determined according to its LOD peak location and surrounding region. The percentage of the phenotypic variance explained by a QTL (R2) was estimated at the highest probability peak. The QTL results were drawn using a custom Perl script (https://github.com/Niuyongchao/Fish_linkage_map). Host resistance level to the complex natural environment was defined as a duration spent in the pathogen-rich environment (the number of days from challenge to morbidity/mortality sampling or final sampling, respectively), which was used as quantitative trait for QTL mapping.

### QTL verification and identification of potential candidate genes related to increased robustness

In order to verify the identified QTL, a closely associated SNP nearest to the QTL peak region (R1-61,252) was validated using Sanger-based genotyping. For this we used offspring from a different mass cross population which was challenged under similar conditions at the same fish farm. Altogether, 255 ‘sensitive’ and 288 ‘robust’ individuals were genotyped using the following primers: forward—5’-TCAGAGCTCAGGTTTAATGGTG-3’ and reverse –5’-TGCCACCGTTGATTTTGGTAG-3’. The resulting genotypes significantly correlated with phenotypes of increased robustness were analyzed using Chi-square test.

Genomic sequences of the QTL region were retrieved to identify the potential causative candidate genes for increased robustness by mapping the corresponding tags of SNPs in QTL regions to the Asian seabass genome. The corresponding gene IDs were obtained from the gene annotation file. Gene functions were collected from literatures searches, BLAST2GO (www.blast2go.org), Uniprot (http://www.uniprot.org), GeneCards (http://www.genecards.org) and OMIM (https://www.omim.org) databases. Pathway information was retrieved from KEGG Automatic Annotation Server (KAAS) (http://www.genome.jp/kegg/kaas/) with Bi-directional Best Hit (BBH) setting [63].

### Detecting mis-assemblies in Asian seabass genome

The flanking sequences of each SNP were mapped onto Asian seabass genome version 2 (v2, contigs/scaffolds level assembly) and version 3 (v3, chromosome-level assembly) [35] using the Burrows-Wheeler Aligner (0.7.10-r789) [64] with the BWA-MEM algorithm. The nucleotide position (bp) of the SNP within the sequences was reordered in the corresponding contigs/scaffolds (v2) and also the reference chromosome (v3). A contig/scaffold was considered as a putative mis-assembly and broken, if the contained SNPs were located in more than one linkage groups. In order to confirm putative mis-assemblies, cleaned reads from two Illumina short-insert fragment libraries (500 bp and 750 bp; 80X coverage) and 90X PacBio reads generated earlier for the published genome assembly [35] were aligned to them by REAPR (Version 1.0.18) [36] to generate read-depth information and identify repetitive regions.

### Supplementary Information

**Additional file 1: Figure S1.** Survival curves of three batches of Asian seabass fingerlings exposed to sea water at typical farm conditions. Two batches (blue and red) were transferred to the farm and exposed to raw sea water during the 28-36 dph period, whereas the third (green) at 56 dph. Losses in the first two batches were more substantial than in the third, indicating the importance of the age of fish at transfer. **Figure S2.** The robustness of families was visualized by analyzing their relative contribution (%) at the beginning vs. the end of experiment. Data from five families are shown: The family produced by brooders Male B & Female A (yellow) shown a substantial 2.5-fold increase in its final relative contribution to the mix in comparison to the initial one, whereas the remaining families either showed decrease (orange and grey) or statistically insignificant change (light blue and dark blue). **Figure S3.** Large numbers of ‘big belly’ bacteria scattered throughout the intestine of infected Asian seabass. In the intestine, there was extensive fulminating granulomatous inflammation with hemorrhage, and with large number of ‘big belly’ bacteria scattered throughout the tissue (green arrowheads).

**Additional file 2: Table S1.** The number of ddRAD sequencing reads for the two Asian seabass brooders and each of their 172 offspring individuals. **Table S2.** Marker IDs, SNP positions and RAD-tag sequences. **Table S3.** Genetic positions of the 3,087 SNP markers in 24 linkage groups of the consensus linkage map of Asian seabass. **Table S4.** Alignment of 19 identified ‘conflict sequences’ to markers on multiple linkage groups. **Table S7.** An additional 100 previously unplaced contigs/scaffolds with a total length of 13.1 Mb were placed onto 22 chromosomes (excluding those corresponding to ASB_LG13 and ASB_LG15) for potential further improvement of the genome published earlier.

**Additional file 3: Table S5.** Six sequences showing mis-matches between the specific LGs and their corresponding chromosomes by checking syntenic relationships of SNPs in each linkage group with its corresponding chromosome. **Table S6.** Summary of all mis-assembled sequences, including four unitigs and 16 scaffolds identified by the ddRAD map.

## Data Availability

Raw sequence data were submitted to the National Center for Biotechnology Information Sequence Read Archive under the BioProject accession number PRJNA890911 (https://dataview.ncbi.nlm.nih.gov/object/PRJNA890911?reviewer=8l57kiogt9g81kllkcplcp22ui).

## Abbreviations

ASB: Asian seabass
ddRAD: Double digest restriction-site associated DNA
ddec: Days during environmental challenge
dph: Days post-hatch
dpt: Days post-transfer
GO: Gene ontology
GS: Genomic selection
GWAS: Genomewide association studies
KEGG: Kyoto Encyclopedia of Genes and Genomes
LG: Linkage group
OD: Logarithm of odds
MAS: Marker-assisted selection
QTL: Quantitative Trait Locus/Loci
SNP: Single nucleotide polymorphism
