# Supplementary material for: Mapping of a major QTL for increased robustness and detection of genome assembly errors in Asian seabass (Lates calcarifer)
Source: BMC Genomics. 2023 Aug 10;24:449. doi: 10.1186/s12864-023-09513-z (PMC10413685; doi:10.1186/s12864-023-09513-z)
Supplement: Supplementary file 1 — Additional file 1: Figure S1. Survival curves of three batches of Asian seabass fingerlings exposed to sea water at typical farm conditions. Two batches (blue and red) were transferred to the farm and exposed to raw sea water during the 28-36 dph period, whereas the third (green) at 56 dph. Losses in the first two batches were more substantial than in the third, indicating the importance of the age of fish at transfer. Figure S2. The robustness of families was visualized by analyzing their relative contribution (%) at the beginning vs. the end of experiment. Data from five families are shown: The family produced by brooders Male B & Female A (yellow) shown a substantial 2.5-fold increase in its final relative contribution to the mix in comparison to the initial one, whereas the remaining families either showed decrease (orange and grey) or statistically insignificant change (light blue and dark blue). Figure S3. Large numbers of ‘big belly’ bacteria scattered throughout the intestine of infected Asian seabass. In the intestine, there was extensive fulminating granulomatous inflammation with hemorrhage, and with large number of ‘big belly’ bacteria scattered throughout the tissue (green arrowheads). [file 12864_2023_9513_MOESM1_ESM.docx]

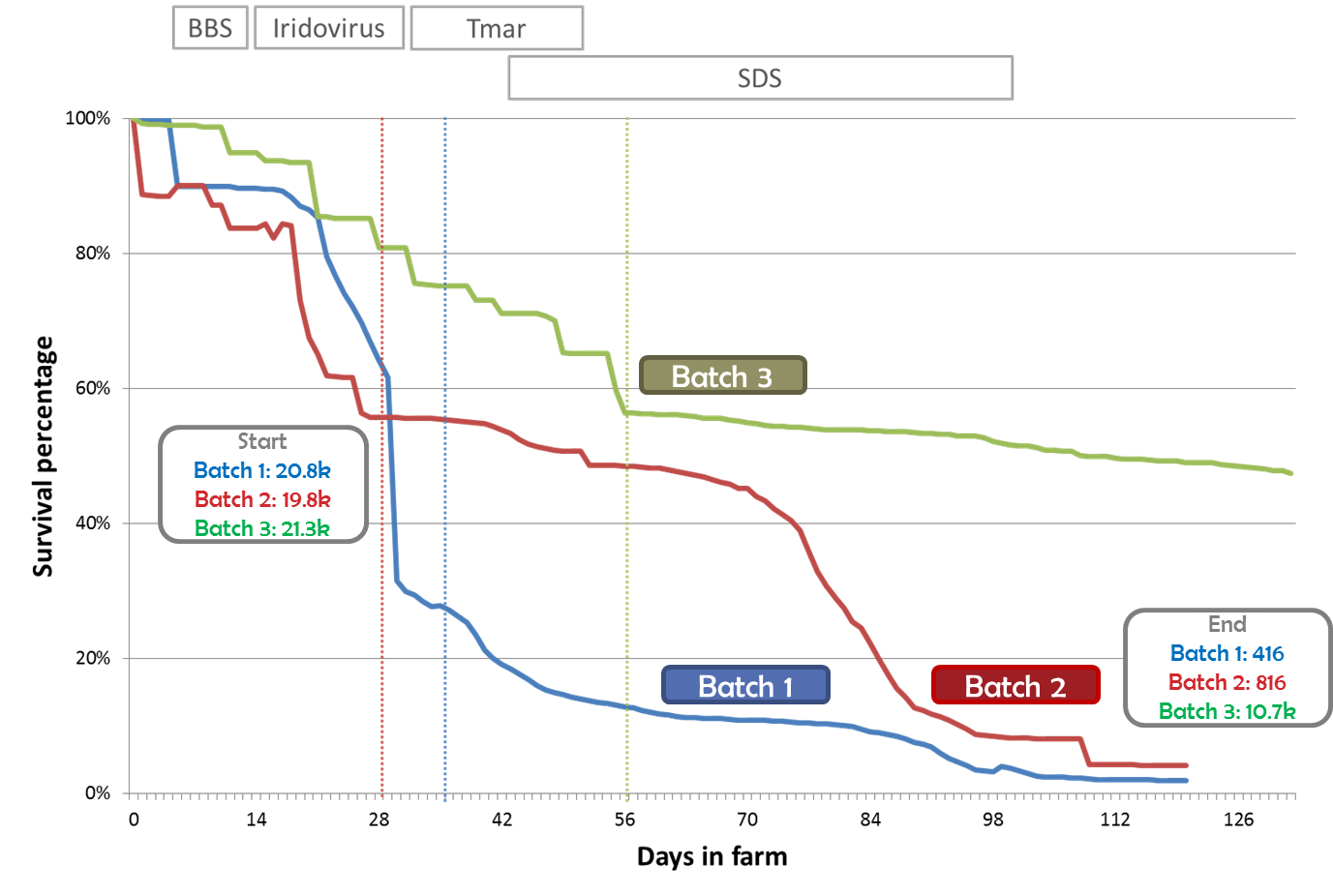


**Age (dph)**

**Figure S1. Survival curves of three batches of Asian seabass fingerlings exposed to sea water at typical farm conditions.** Two batches (blue and red) were transferred to the farm and exposed to raw sea water during the 28-36 dph period, whereas the third (green) at 56 dph. Losses in the first two batches were more substantial than the third, indicating the importance of the age of fish at transfer.


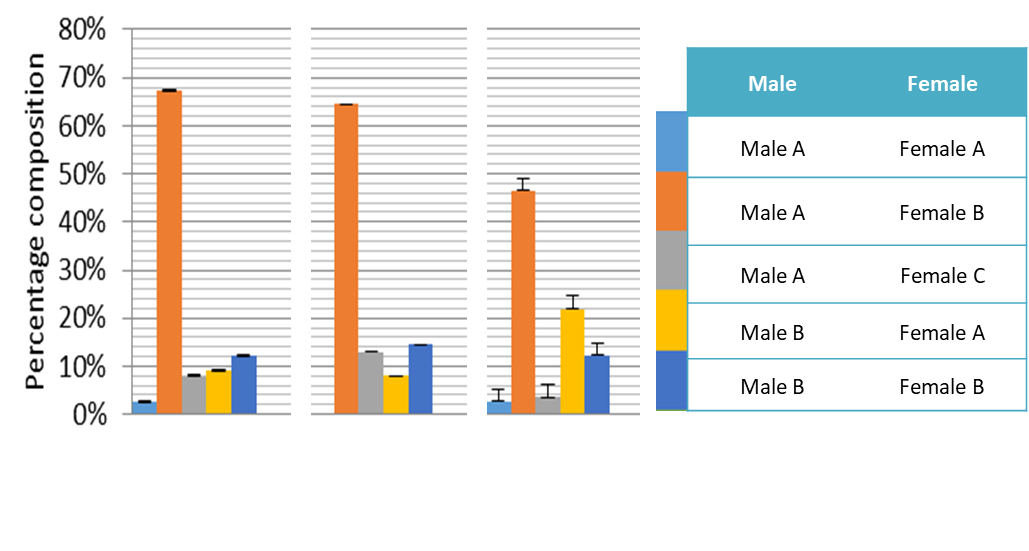


Contributions

Initial Sensitive Robust

**Figure S2. The robustness of families was visualized by analyzing their relative contribution at the beginning and the end of the experiment.** Data from five families are shown: The family with brooders Male B & Female A (yellow) shown a substantial 2.5-fold increase in its final relative contribution to the survivors (robust) in comparison to the initial one, whereas the remaining families either showed decrease (orange and grey) or statistically insignificant change (light blue and dark blue).


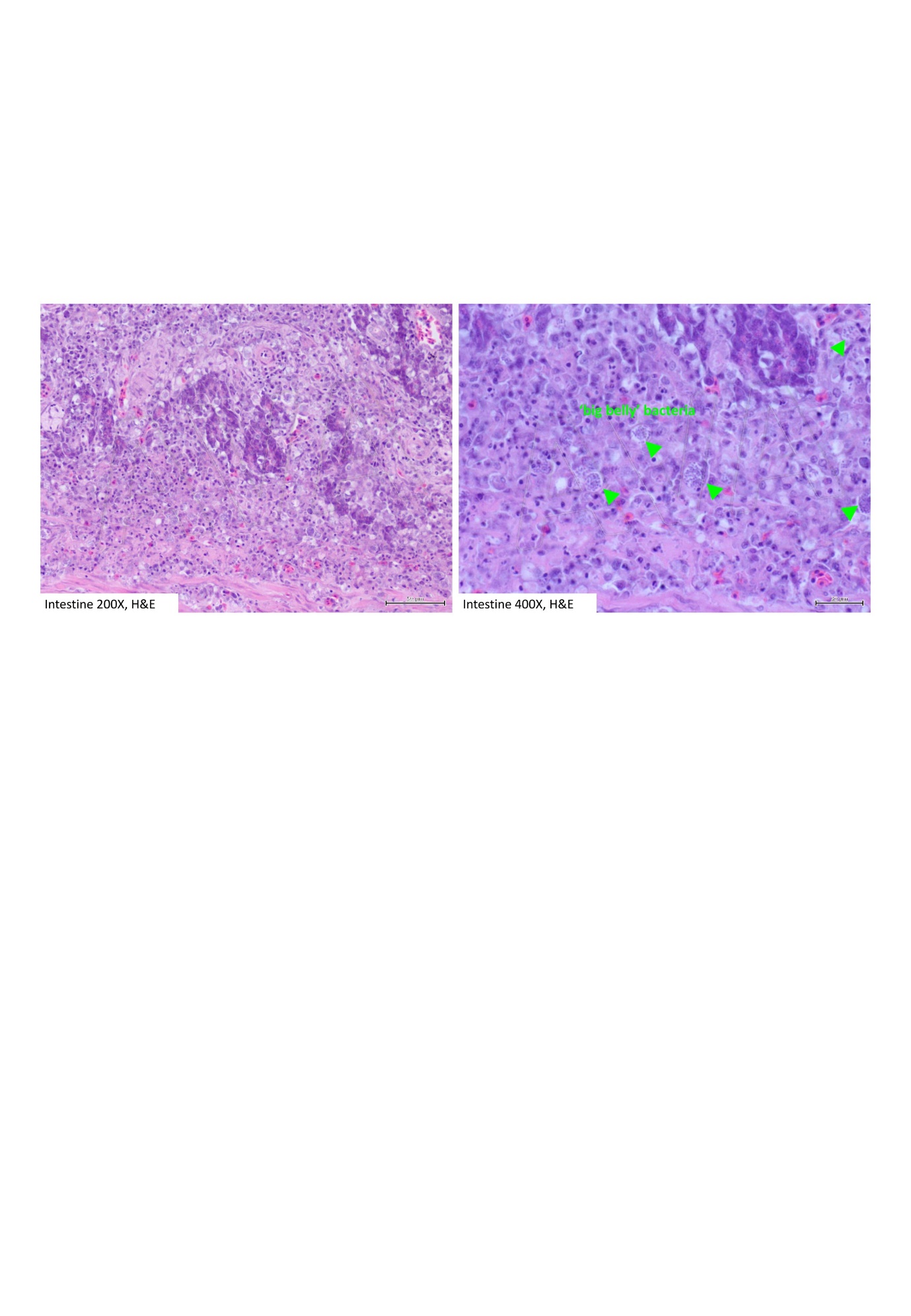


**Figure S3. Large numbers of ‘big belly’** **bacteria scattered throughout the intestine of infected Asian seabass.** In the intestine, there was extensive fulminating granulomatous inflammation with hemorrhage, and with large number of ‘big belly’ bacteria scattered throughout the tissue (green arrowheads).
