# Supplementary material for: Mapping of a major QTL for increased robustness and detection of genome assembly errors in Asian seabass (Lates calcarifer)
Source: BMC Genomics. 2023 Aug 10;24:449. doi: 10.1186/s12864-023-09513-z (PMC10413685; doi:10.1186/s12864-023-09513-z)
Supplement: Supplementary file 3 — Additional file 3: Table S5. Six sequences showing mis-matches between the specific LGs and their corresponding chromosomes by checking syntenic relationships of SNPs in each linkage group with its corresponding chromosome. Table S6. Summary of all mis-assembled sequences, including four unitigs and 16 scaffolds identified by the ddRAD map. [file 12864_2023_9513_MOESM3_ESM.docx]

**Table S5.** Six sequences showing mis-matches between the specific LGs and their corresponding chromosomes by checking syntenic relationships of SNPs in each linkage group with its corresponding chromosome

| LG ID (total mapped markers) | Corespounding chromosome (V3) | Inconsistant mapped markers | ID of inconsistant mapped chromosome | Position on chromosome (bp) | Mapped scaffolds/unitigs | Position on scaffolds/unitigs (bp) |
| --- | --- | --- | --- | --- | --- | --- |
| LG12 (179) | ASB_LG12 | R1-27801 | ASB_LG8 | 10042850 | **@unitig_4383\|quiver** | 1861403 |
|  |  | R1-84736 | ASB_LG8 | 10025489 | @unitig_4383\|quiver | 1878764 |
|  |  | R1-50437 | ASB_LG20 | 1868914 | **@unitig_4480\|quiver** | 669670 |
|  |  | R1-71963 | ASB_LG20 | 1886416 | @unitig_4480\|quiver | 687172 |
|  |  | R1-32690 | ASB_LG20 | 2385789 | @unitig_4480\|quiver | 1186545 |
|  |  | R1-9022 | ASB_LG9 | 4421954 | **scaffold_74** | 348543 |
|  |  | R1-34655 | ASB_LG9 | 4428240 | scaffold_74 | 354829 |
|  |  | R1-88342 | ASB_LG9 | 4500941 | scaffold_74 | 427530 |
| LG23 (103) | ASB_LG23 | R1-64847 | ASB_LG8 | 14315729 | **@unitig_4955\|quiver** | 2054320 |
| LG14 (86) | ASB_LG14 | R1-62654 | ASB_LG9 | 20764709 | **@unitig_2144\|quiver** | 5976 |
|  |  | R1-8094 | ASB_LG9 | 20852288 | @unitig_2144\|quiver | 93555 |
|  |  | R1-78363 | ASB_LG9 | 20879766 | @unitig_2144\|quiver | 121033 |
|  |  | R1-90577 | ASB_LG9 | 20888619 | @unitig_2144\|quiver | 129886 |
|  |  | R1-48207 | ASB_LG15 | 18373342 | **scaffold_18** | 14083209 |
|  |  | R1-65545 | ASB_LG15 | 18682459 | scaffold_18 | 13774092 |
|  |  | R1-52116 | ASB_LG15 | 18718797 | scaffold_18 | 13737754 |
|  |  | R1-54340 | ASB_LG15 | 18888711 | scaffold_18 | 13567840 |
|  |  | R1-23452 | ASB_LG15 | 19423674 | scaffold_18 | 13032877 |
|  |  | R1-73693 | ASB_LG15 | 19483862 | scaffold_18 | 12972689 |
|  |  | R1-6127 | ASB_LG15 | 19678770 | scaffold_18 | 12777781 |
|  |  | R1-14042 | ASB_LG15 | 19726703 | scaffold_18 | 12729848 |
|  |  | R1-57119 | ASB_LG15 | 19751350 | scaffold_18 | 12705201 |
|  |  | R1-40338 | ASB_LG15 | 19880398 | scaffold_18 | 12576153 |
|  |  | R1-1586 | ASB_LG15 | 19989269 | scaffold_18 | 12467282 |
|  |  | R1-25646 | ASB_LG15 | 19994868 | scaffold_18 | 12461683 |
|  |  | R1-4302 | ASB_LG15 | 20377190 | scaffold_18 | 12079361 |
|  |  | R1-16698 | ASB_LG15 | 20479663 | scaffold_18 | 11976888 |
|  |  | R1-83576 | ASB_LG15 | 20770338 | scaffold_18 | 11686213 |
|  |  | R1-87540 | ASB_LG15 | 20806399 | scaffold_18 | 11650152 |

**Table S6.** Summary of all mis-assembled sequences, including four unitigs and 16 scaffolds identified by the ddRAD map.

| Mis-assembled sequences ID | No. of total mapped SNPs | Mapped linkage groups (No. of markers) | ID of mapped chromosomes (No. of markers) |
| --- | --- | --- | --- |
|  |  |  |  |
| unitig_2144\|quiver | 4 | LG14 (4) | ASB_LG9 (4) |
| unitig_4383\|quiver | 8 | LG12 (2), LG8 (6) | ASB_LG8 (8) |
| unitig_4480\|quiver | 7 | LG12 (3), LG20 (4) | ASB_ LG20 (7) |
| unitig_4955\|quiver | 4 | LG8 (3), LG23 (1) | ASB_LG8 (4) |
| scaffold_1 | 17 | LG6 (11); LG9 (6) | ASB_LG6 (11); ASB_ LG9 (6) |
| scaffold_16 | 8 | LG16_22 (1); LG6 (7) | ASB_LG16_22 (1); ASB_LG6 (7) |
| scaffold_18 | 87 | LG10 (12), LG14 (16), LG15 (59) | ASB_LG10 (12), ASB_LG15 (75) |
| scaffold_21 | 7 | LG1 (1), LG9 (6) | ASB_LG1 (1), ASB_LG9 (6) |
| scaffold_28 | 31 | LG21 (25), LG11 (6) | ASB_LG21 (25), ASB_LG11 (6) |
| scaffold_46 | 5 | LG1 (1), LG9 (4) | ASB_LG1 (1), ASB_LG9 (4) |
| scaffold_48 | 18 | LG13 (6), LG24 (12) | ASB_LG13 (6), ASB_LG24 (12) |
| scaffold_63 | 24 | LG6 (21), LG5 (3) | ASB_LG6 (21), ASB_LG5 (3) |
| scaffold_66 | 24 | LG10 (22), LG6 (2) | ASB_LG10 (22), ASB_LG6 (2) |
| scaffold_74 | 6 | LG12 (4), LG9 (2) | ASB_LG12 (4), ASB_LG9 (2) |
| scaffold_77 | 5 | LG1 (3), LG23 (2) | ASB_LG1 (3), ASB_LG23 (2) |
| scaffold_83 | 10 | LG12 (6), LG16_22 (4) | ASB_LG12 (6), ASB_LG16_LG22 (4) |
| scaffold_84 | 3 | LG12 (1), LG 4 (2) | ASB_LG12 (1), ASB_LG 4 (2) |
| scaffold_87 | 3 | LG16_22 (2), LG23 (1) | ASB_LG16_LG22 (2), ASB_LG23 (1) |
| scaffold_90 | 12 | LG7_1 (7), LG23 (5) | ASB_LG7_1 (7), ASB_LG23 (5) |
| scaffold_94 | 13 | LG12 (11), LG8 (2) | ASB_LG12 (11), ASB_LG8 (2) |
